# Antifibrotic Effect of Smad Decoy Oligodeoxynucleotide in a CCl_4_-Induced Hepatic Fibrosis Animal Model

**DOI:** 10.3390/molecules23081991

**Published:** 2018-08-10

**Authors:** Mi-Gyeong Gwon, Jung-Yeon Kim, Hyun-Jin An, Woon-Hae Kim, Hyemin Gu, Min-Kyung Kim, Sok Cheon Pak, Kwan-Kyu Park

**Affiliations:** 1 Department of Pathology, College of Medicine, Catholic University of Daegu, 33, Duryugongwon-ro 17-gil, Nam-gu, Daegu 42472, Korea; daldy88@cu.ac.kr (M.-G.G.); jy1118@cu.ac.kr (J.-Y.K.); ahj119@cu.ac.kr (H.-J.A.); kimwoonhae@cu.ac.kr (W.-H.K.); guhm1207@cu.ac.kr (H.G.); 2 Department of Pathology, Dongguk University School of Medicine, Gyeongju 38066, Korea; minkyungk76@naver.com; 3 School of Biomedical Sciences, Charles Sturt University, Panorama Avenue, Bathurst, NSW 2795, Australia; spak@csu.edu.au

**Keywords:** liver fibrosis, Smad, decoy, oligodeoxynucleotide, CCl_4_

## Abstract

Hepatic fibrosis is the wound-healing process of chronic hepatic disease that leads to the end-stage of hepatocellular carcinoma and demolition of hepatic structures. Epithelial–mesenchymal transition (EMT) has been identified to phenotypic conversion of the epithelium to mesenchymal phenotype that occurred during fibrosis. Smad decoy oligodeoxynucleotide (ODN) is a synthetic DNA fragment containing a complementary sequence of Smad transcription factor. Thus, this study evaluated the antifibrotic effects of Smad decoy ODN on carbon tetrachloride (CCl_4_)-induced hepatic fibrosis in mice. As shown in histological results, CCl_4_ treatment triggered hepatic fibrosis and increased Smad expression. On the contrary, Smad decoy ODN administration suppressed fibrogenesis and EMT process. The expression of Smad signaling and EMT-associated protein was markedly decreased in Smad decoy ODN-treated mice compared with CCl_4_-injured mice. In conclusion, these data indicate the practicability of Smad decoy ODN administration for preventing hepatic fibrosis and EMT processes.

## 1. Introduction

The liver is the main organ of immense complexity, responsible for the metabolism of drugs and toxic chemicals [1]. Chronic liver diseases affecting hundreds of millions of people globally are associated with a developed progress of hepatocellular carcinoma and hepatic fibrosis [2]. Hepatic fibrosis is a consequence of wound-healing responses of the liver that is caused by chronic liver injuries such as alcoholic, viral, and autoimmune hepatitis [3]. Regardless of causes, direct and sustained hepatic injury induces a persistent inflammatory response and excessive deposition of extracellular matrix (ECM) in the perisinusoidal space of Disse, leading to the progression of hepatic fibrosis [4]. Hepatic fibrogenesis affects both hepatocytes and nonparenchymal cells such as hepatic stellate cells (HSCs), which are prerequisite for preserving an entire liver structure and function [5,6].

Hepatocytes and HSCs as the major source of myofibroblasts in injured livers play an important role in the progress of liver inflammation and development of hepatic fibrosis. Any chronic form of hepatic injury can result in the transformation of hepatocytes into mesenchymal cells by an epithelial–mesenchymal transition (EMT) process. EMT is a dynamic program in which fully differentiated epithelial cells undergo a phenotypic change, resulting in loss of marker proteins such as E-cadherin and zonula occludens-1 (ZO-1), and acquisition of mesenchymal characteristics such as α-SMA, vimentin, matrix metalloproteinase (MMP)-2, MMP-9, and collagens [7,8]. In addition, following damage to the epithelial cells, bile-duct epithelium and hepatocytes released the profibrogenic cytokine that further activates HSCs [9,10]. The activated HSCs lead to hepatic fibrogenesis by excessive production of ECM components, such as type I collagen and fibronectin [8,9].

Carbon tetrachloride (CCl_4_), a classic hepatotoxic agent, is commonly used to induce liver injury in experimental animal models to examine the pathogenesis of fibrosis and hepatic cirrhosis [1]. This liver injury is ascribed to inflammation originating from CCl_4_-derived trichloromethylperoxy free-radical formation in the liver [11]. CCl_4_ treatment induces centrilobular necrosis that causes a wound-healing response that starts with the recruitment of inflammatory and phagocytic cells in liver necrotic zones, then accumulation of ECM, and the release of fibrotic cytokines. Finally, continued hepatic inflammatory responses provoked by prolonged CCl_4_ administration are believed to induce hepatic fibrosis, cirrhosis, and hepatocellular carcinoma [12,13,14].

Many studies have identified that a variety of cytokines and growth factors, including transforming growth factor-β1 (TGF-β1), epidermal growth factor (EGF), and hepatocyte growth factor (HGF), participate in the EMT process [7,15,16]. TGF-β1/Smad signaling has been reported as a mechanism leading to hepatic fibrosis. TGF-β1 activates Smad-dependent and Smad-independent pathways to display its biological activities. For Smad-dependent pathways, TGF-β1 exerts diverse biological activities via its intracellular mediators Smad2 and Smad3, and is negatively regulated by an inhibitory mediator, Smad7 [17]. Smads have been also identified to interact with other pathways, such as the MAPK and NF-κB signaling pathways [18]. However, these cellular-signaling processes still remain unclear in the hepatocytes. Therefore, a recent review concentrates on the regulatory mechanisms and functional role of TGF-β1/Smad pathways during the progression of hepatic fibrosis [19]. Moreover, the development of treatment targeting TGF-β1/Smad pathways is still in its infancy [20].

Decoy oligodeoxynucleotide (ODN) is a synthetic short DNA segment containing a consensus-binding sequence that competitively combines with a target-transcription factor [21]. As a result, the decoy ODN binds to the specific transcription factor and inhibits gene expression by preventing the upregulation of involved genes. We previously demonstrated that inhibition of Smad and Sp1 by the decoy ODN strategy prevented renal fibrosis in mice via inhibition of the production of cytokines related to fibrosis and EMT [22]. Additionally, we demonstrated the antifibrotic effect of the NF-κB decoy ODN in a hepatic fibrosis animal model [23]. However, the effect of Smad ODN on hepatic fibrosis in hepatocytes has not been reported. Therefore, we investigated the antifibrotic effect of Smad decoy ODN on hepatic fibrosis by regulating a Smad-signaling pathway and EMT process, using CCl_4_-induced hepatic fibrosis.

## 2. Results

### 2.1. Transfection Efficiency and DNA-Binding Activity of Smad Decoy ODN in the CCl_4_-Treated Mouse Liver

**Figure 1:**
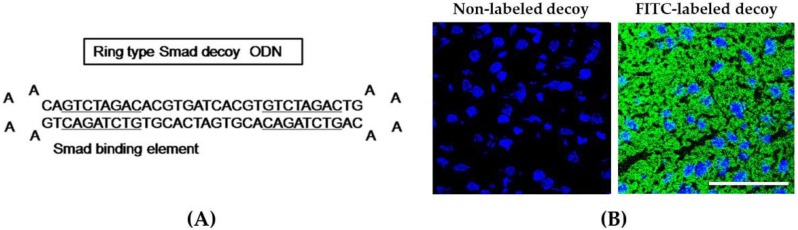
Synthesis of Smad decoy oligodeoxynucleotide (ODN) and transfection of Smad decoy ODN into mouse liver. (**A**) Design of ring-type Smad decoy ODN including GTCTAGAC, which is the consensus sequence for the Smad-binding element; (**B**) immunofluorescence image of the transfer effect of Smad decoy ODN in liver of mice. The image on the left is the result of administration of nonlabeled ODN, and on the image on the right, green fluorescence represented successful transfection into mouse liver. Scale bar, 50 μm.

We designed the hair-pin structure Smad decoy ODN and synthesized the double-stranded decoy ODN that contains the sequence Smad binding element (Figure 1A). To identify the successful transfer of Smad decoy ODN, we analyzed the transfection efficiency of the FITC-labeled ODN using fluorescence microscopy. FITC-labeled Smad ODNs, which were administered intravenously and detected by fluorescence, were shown in the cytoplasm and nucleus of liver cells (Figure 1B). These results indicate that Smad decoy ODN was successfully transfected into a mouse liver.

### 2.2. Smad Decoy ODN Attenuated Morphological Changes in CCl_4_-Induced Hepatic Fibrosis

**Figure 2:**
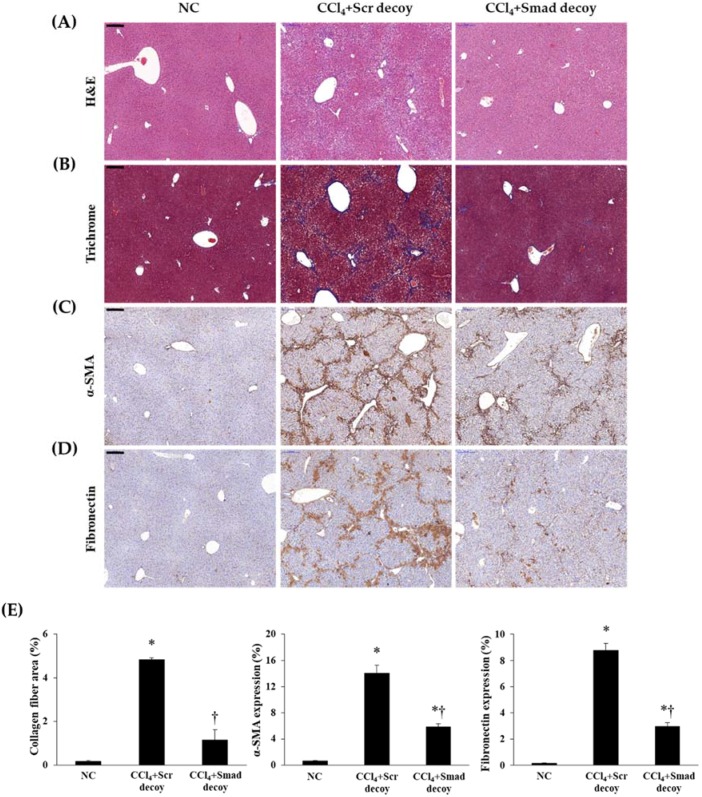
Smad decoy ODN attenuated morphological changes in carbon tetrachloride (CCl_4_)-induced hepatic fibrosis in mice. (**A**) Paraffin-embedded liver section stained with hematoxylin and eosin (H&E) staining; (**B**) Masson’s trichrome staining; (**C**) α-SMA and (**D**) fibronectin were detected by immunohistochemical staining; (**E**) Quantification of collagen, α-SMA, and fibronectin expressions. Original Scale bar = 200 μm. * *p* < 0.05 compared to normal control group; † *p* < 0.05 compared to the CCl_4_+Scr group.

CCl_4_ administration induced centrilobular necrosis, proliferation of parenchymal cells and nonparenchymal cells, fibrosis, and accumulation of ECM [19]. To identify the antifibrotic effect of Smad decoy ODN in hepatic fibrogenesis, we used a CCl_4_-induced hepatotoxic model. To show histological change, we performed both hematoxylin and eosin (H&E) staining (Figure 2A) and Masson’s trichrome staining (Figure 2B). The basic lobular architecture was well preserved with a portal vein in the normal control group. Cellular inflammation, ballooning changes of hepatocytes, and lobular necrosis had developed around the sinusoids in CCl_4_-treated mouse. These changes were significantly attenuated by Smad decoy ODN treatment. Additionally, Smad decoy ODN was able to prevent the accumulation of collagen caused by CCl_4_-induced liver damage. Taken together, these data indicate that Smad decoy ODN suppressed morphological changes and collagen accumulation in the CCl_4_-injected mice.

### 2.3. Smad Decoy ODN Suppressed ECM Accumulation and EMT Process in CCl_4_-Induced Hepatic Fibrosis

**Figure 3:**
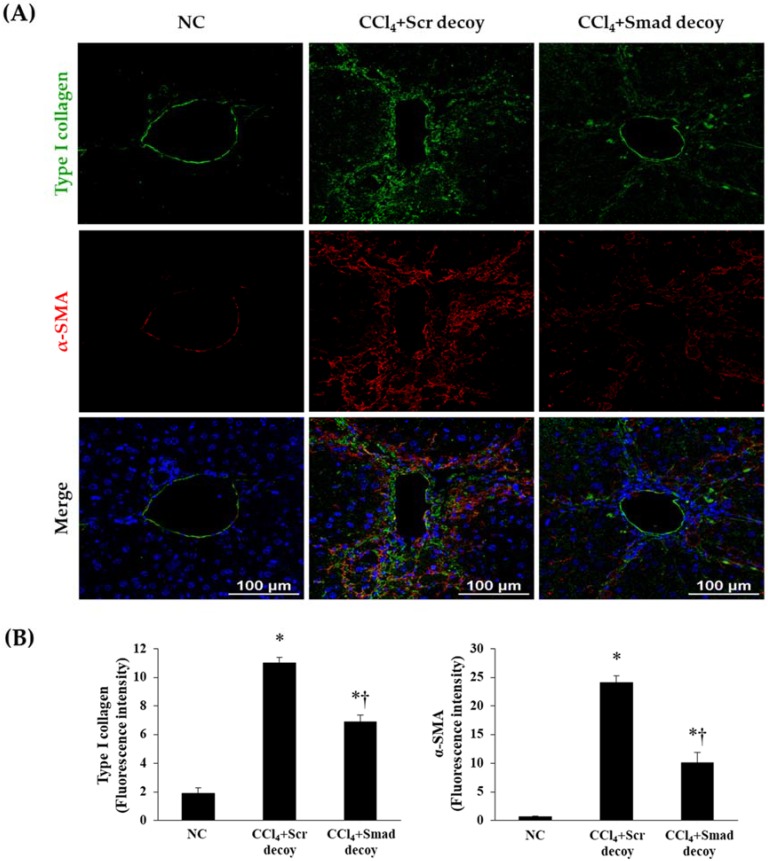
Expression of extracellular matrix (ECM) decreased by Smad decoy ODN in CCl_4_-induced hepatic fibrosis in mice. (**A**) Immunofluorescence double staining for type I collagen (green) and α-SMA (red) showed that Smad decoy ODN administration suppressed ECM deposition around of portal vein. The nuclei were stained with Hoechst. (**B**) Quantification of type I collagen and α-SMA fluorescence intensity. Scale bar = 100 μm. * *p* < 0.05 compared to normal control group; † *p* < 0.05 compared to the CCl_4_+Scr group.

Following liver injury, hepatic stellate cells undergo phenotypic changes, which leads to increased deposition of ECM proteins, such as α-SMA, fibronectin, and type I collagen in the hepatic sinusoid [23]. Therefore, we investigated the effects of Smad decoy ODN on hepatic fibrogenesis and ECM accumulation by immunohistochemical and immunofluorescent staining in CCl_4_-induced hepatic fibrosis. The expression of α-SMA as a myofibroblasts marker was obviously elevated in CCl_4_-injured mice compared with normal mice. However, the administration of Smad decoy ODN resulted in the downregulation of α-SMA expression (Figure 2C). The expression of fibronectin within the liver sinusoid increased in CCl_4_-induced fibrosis mice, whereas administration of Smad decoy suppressed fibronectin expression (Figure 2D). The effect of Smad decoy ODN on CCl_4_-induced fibrosis in the liver was further investigated by immunofluorescent staining. The expression of α-SMA and type I collagen was increased in the liver tissue of the CCl_4_-injured mice, but this increase was inhibited by Smad decoy ODN (Figure 3).

**Figure 4:**
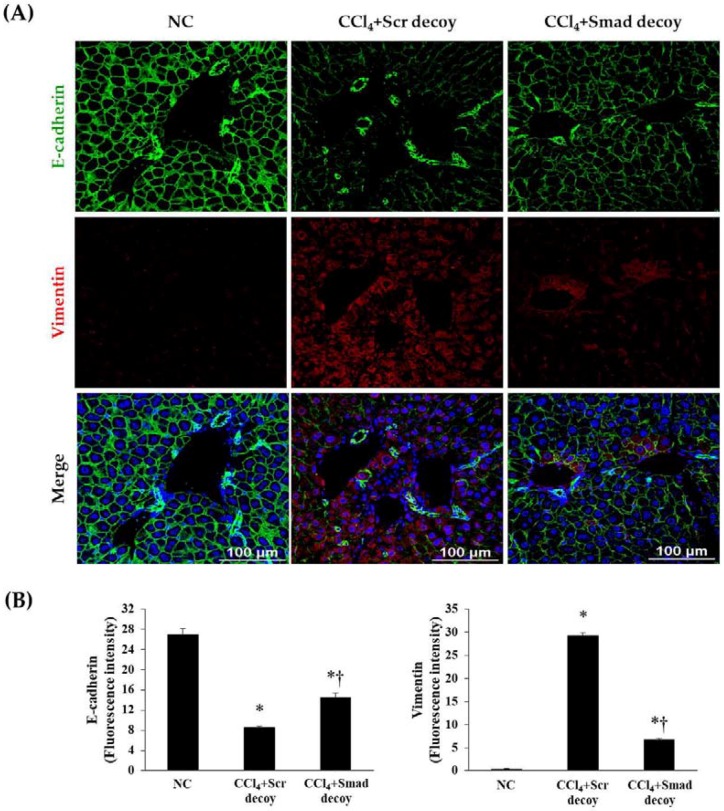
Effect of Smad decoy ODN on the expression of epithelial to mesenchymal transition (EMT). (**A**) Representative immunofluorescence staining showed that Smad ODN administration increased expression of E-cadherin (green) and decreased expression of vimentin (red) around of portal vein of hepatic fibrosis in mice. To counterstain, the nuclei were labeled with Hoechst 33342 (blue). (**B**) Quantification of E-cadherin and vimentin fluorescence intensity. Scale bar = 100 μm. * *p* < 0.05 compared to normal control group; † *p* < 0.05 compared to the CCl_4_+Scr group.

During the EMT in hepatic fibrosis, intercellular junctions of epithelial cells are disrupted by downregulation of E-cadherin, which is confirmed by increase of mesenchymal phenotype, including vimentin [24]. As shown in Figure 4, the expression of E-cadherin in immunofluorescence was decreased after CCl_4_ injury compared with normal control, and vimentin showed the opposite result. However, Smad decoy ODN administration suppressed this expression change in the fibrotic liver. These findings show that Smad decoy ODN has antifibrotic characteristics through the downregulation of ECM expression and disruption of EMT process following liver injury.

### 2.4. Smad Decoy ODN Inhibited Fibrotic Genes by Regulating Smad-Dependent Signaling Pathway in CCl_4_-Induced Hepatic Fibrosis

**Figure 5:**
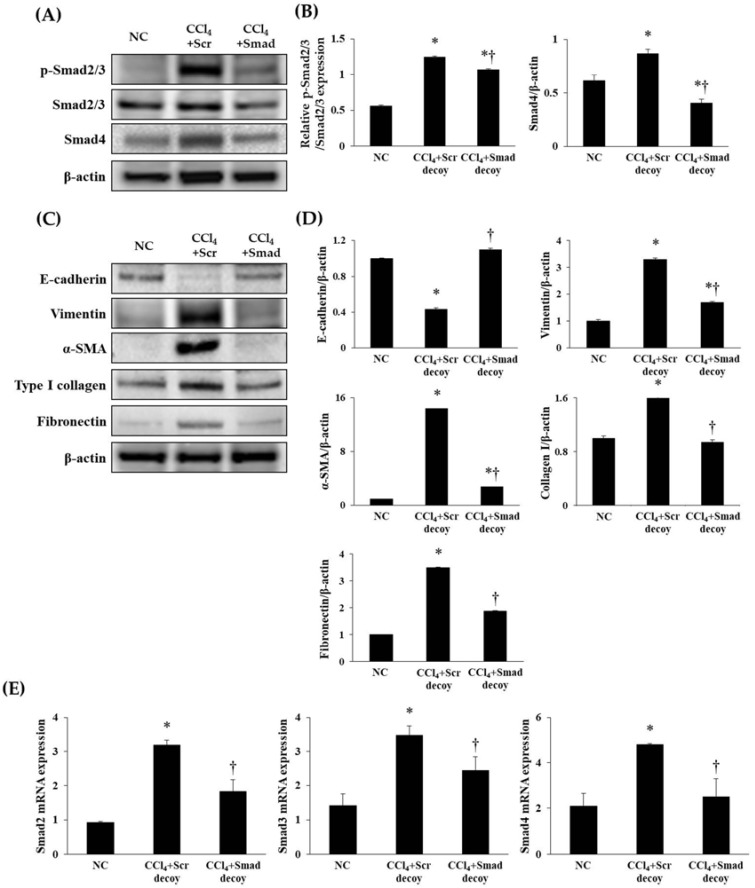
Synthetic Smad decoy ODN significantly suppressed fibrotic genes and ECM proteins by blocking the Smad-dependent signaling pathway. (**A**) Western blot data showed that Smad decoy ODN suppressed the expression of p-Smad2/3 and Smad4. All samples were loaded equal volumes, which was confirmed by loading GAPDH together; the results are representative of three independent experiments; (**B**) The expression levels of the protein from quantification of these images by Image J. (**C**) The expressions of E-cadherin, vimentin, α-SMA, type I collagen, and fibronectin as the representative of three independent experiments; (**D**) quantification of ECM proteins; and (**E**) qRT-PCR analysis that showed that Smad decoy ODN inhibited the mRNA expression of Smad2, Smad3, and Smad4. * *p* < 0.05 compared to normal control group; † *p* < 0.05 compared to the CCl_4_+Scr group.

TGF-β1/Smad signaling has been demonstrated to be a crucial mediator in EMT and subsequent fibrosis. A rapid relocation of Smad proteins by TGF-β1 signaling is followed by transcription of the fibrogenesis gene [25]. To investigate the regulation of Smad decoy ODN on the TGF-β1 downstream signaling pathway, we investigated the expression of p-Smad2/3 and Smad4 by Western blotting. The expressions of p-Smad2/3 and Smad4 were markedly increased in hepatic fibrosis mice compared with normal mice (*p* < 0.05, Figure 5A,B). However, injection of Smad decoy ODN attenuated CCl_4_-induced fibrotic mediator production through suppression of binding Smad transcription factor in the DNA-binding site. In addition, quantitative real-time polymerase chain reaction (qRT-PCR) data showed that Smad decoy ODN inhibits the Smad signaling pathway (Figure 5E). Next, fibrogenesis and EMT expression levels in the fibrotic liver were measured by Western blot (Figure 5C,D). Western blot results showed that the expression of vimentin, α-SMA, fibronectin, and type I collagen increased in fibrotic liver compared to normal control mice (*p* < 0.05). However, Smad ODN administration decreased the tissue vimentin, α-SMA, type I collagen, and fibronectin levels. On the other hand, the CCl_4_ inhibited E-cadherin expression, while a relative amount of E-cadherin was preserved in Smad decoy ODN-treated livers. These results indicate that Smad decoy ODN inhibits hepatic EMT and fibrosis via blocking the TGF-β1-stimulted Smad signaling pathway in CCl_4_-induced hepatic fibrosis.

## 3. Discussion

Hepatic fibrosis is a complicated pathological process of several chronic liver diseases. Many studies have underlined the potentiality of the Smad-signaling group participation in the pathogenesis of hepatic fibrosis and carcinogenesis (fibrocarcinogenesis) [26,27]. Smad proteins play a crucial role in the transduction of receptor signals to target genes in the nucleus [6,28].

Decoy ODN is a double-stranded DNA segment containing a specific transcription factor-binding element [21]. Some studies reported that the induction of synthetic decoy ODN with high attraction for their target transcription factors into peculiar cells. A previous study demonstrated that NF-κB decoy ODN inhibited the EMT process in mice of CCl_4_-induced hepatic fibrosis [23]. Park et al. [29] reported the effect of ring-type decoy ODNs on CCl_4_-induced hepatic fibrosis. Their study showed that a ring-type Sp1 decoy ODN suppressed the level of cytokines, TGF-β1 downstream-target genes, and hepatic fibrosis. In addition, Kim et al. [6] also investigated the role of synthetic TGF-β1/Smad ODN in liver cirrhosis. This study showed that the dual-function ODN effectively blocked the mRNA expression of TGF-β1 and binding of Smad transcription factor. Therefore, the effect of each ODN has not been demonstrated. Thus, in the current study, we synthesized Smad decoy ODN and investigated the effect of Smad decoy ODN on hepatic fibrosis. The Smad decoy ODN used in this study was a synthesized double-stranded ODN containing the consensus Smad-binding element (GTCTAGAC) that binds with the Smad 2/3/4 complex. In our previous study, we demonstrated that synthetic decoy ODN was effectively transfected into hepatocytes [6,23]. On the basis of the results of our previous study, we investigated the effect of Smad decoy ODN on CCl_4_-induced hepatic fibrosis. With this aim in mind, Smad decoy ODN was transfected into liver hepatocytes.

The CCl_4_-induced hepatic fibrosis model is commonly used for antifibrotic research, with many studies showing that CCl_4_, a hepatotoxic agent, activated the TGF-β1/Smad-signaling pathway and led to an accumulation of ECM [23,30]. Increased TGF-β1 initiated intracellular signaling by binding TGF-β receptor type II and TGF-β1, then stimulating TGF-β receptor type I kinase, resulting in the activation of the downstream-signaling pathway [31,32]. Following binding of TGF-β1 to receptors, Smad2 and Smad3 were phosphorylated by TGF-β receptors and its complex with Smad4. This transcription-factor complex was translocated to the nucleus. Complexes of p-Smad2/3 and Smad4 in the nucleus can regulate the transcription of the fibrous gene [33]. Thus, to identify the antifibrotic effect of Smad decoy ODN, we used a CCl_4_ hepatotoxic animal model. Consistent with previous study, our research shows that the CCl_4_ hepatotoxic agent accumulated ECM components and activated TGF-β1 signaling. In addition, the expression levels of p-Smad2/3 and Smad4 were increased by CCl_4_, and Smad decoy ODN decreased them. Fagone et al. [34] identified that genes of hepatic fibrosis were significantly increased in activated HSCs. According to this study, type I collagen and fibronectin were biomarkers for hepatic fibrosis. In the present study, the hepatotoxic agent CCl_4_ stimulated increased expression of ECM proteins and activated Smad-dependent signaling. However, Smad decoy ODN decreased the expression of type I collagen, α-SMA, and fibronectin. It means that CCl_4_ administration induced activation of HSCs and induction of fibrogenesis.

The Smad-dependent signaling response was correlated with the EMT process during hepatic fibrogenesis. In addition, Sung et al. [22] demonstrated that inhibition of Smad signaling attenuated the EMT process and accumulation of ECM in renal fibrosis. Some research also suggested that chronic hepatic injury resulted in the transformation of hepatocytes into myofibroblasts by the EMT process [35]. During EMT, symptoms such as increased migratory capacity, invasiveness, enhanced resistance to apoptosis, and greatly increased ECM production occur. EMT has been classified into three dissimilar biological subtypes built on biological circumstances. The type 2 EMT, classified as organ fibrosis, is associated with organ repair and is involved in secondary morphologic change of epithelial or endothelial cells to resident mesenchymal or fibroblast cells in response to persistent inflammation. These processes lead to the loss of epithelial-marker proteins and acquisition of mesenchymal characteristics [8]. In the present study, the epithelial markers decreased and myofibroblast markers increased by CCl_4_ administration. It suggested that hepatocytes changed into myofibroblasts via the EMT process.

However, this result can be interpreted differently. For example, loss of E-cadherin may have been the result of CCl_4_-induced hepatocyte injury, and proliferation of myofibroblasts may have occurred in response to hepatocyte-secreted cytokines. In addition, several studies investigated that there is a contradiction in the EMT process [36]. The controversy surrounding the EMT process means that it has become one of the most debated topics in hepatic fibrosis study today [37]. In the current study, we only investigated the antifibrotic effect of Smad decoy ODN and observed the EMT processes in an animal model of hepatic fibrosis. Therefore, to support the inhibition effect of Smad decoy ODN on the EMT process, more research is needed.

In summary, this study confirmed that Smad decoy ODN inhibited hepatic fibrosis by blocking the TGF-β1/Smad signaling pathway, which was activated by CCl_4_ administration. CCl_4_-treated mice induced inflammation response and hepatic failure such as accumulation of ECM, centrilobular necrosis, activation of fibrotic genes, and EMT processes. However, effective transfection of Smad decoy ODN attenuated immune responses and pathophysiological changes in the liver. But, in our study, only the histological examination was carried out, and thus it is considered that further study on the expression level of cytokine is needed. In addition, Smad decoy ODN suppressed EMT processes and the production of ECM proteins in CCl_4_-induced hepatic fibrosis. Therefore, these results indicate that Smad decoy ODN is able to protect the liver against hepatic injury.

Taken together, our results demonstrate that Smad decoy ODN suppressed Smad-mediated hepatic fibrosis by blocking the Smad signaling pathway and reducing EMT processes. Given this fact, Smad decoy ODN gene therapy might provide a new therapeutic strategy to prevent hepatic fibrosis. However, more studies are needed to further determine the relationship between the therapeutic use of Smad signaling and hepatic fibrosis to chronic hepatic diseases.

## 4. Materials and Methods 

### 4.1. Synthesis of Ring-Type Smad Decoy ODNs

Decoy ODNs were synthesized by Macrogen (Seoul, Korea). Smad and scrambled (Scr) decoy ODN sequences used are listed below in Table 1 (consensus sequence is underlined).

These structures were annealed for 6 h, while temperature was decreased from 80 °C to 25 °C. These decoy ODNs were predicted to form a hair-pin structure (Figure 1). To obtain a covalent ligation for ring-type decoy ODN molecules, each decoy ODN was mixed with T4 ligase (Takara Bio, Otsu, Japan) and incubated at 16 °C for 18 h.

### 4.2. Animal Models and Smad Decoy ODN Transfection

Animal protocols were approved by the Institutional Animal Care and Use Committee of the Catholic University of Daegu (EXP-IRB number: 2014-0001-CU-AEC-04-A). Male C57BL/6 mice (6 weeks old, 20–22 g; Samtako, Daejeon, South Korea) were housed in a room with controlled humidity and temperature, and a 12 h light–dark cycle. To examine the in vivo transfection efficiency of synthetic Scr ODN, Smad decoy ODN and FITC-labeled Smad decoy ODN were injected into mice intravenously (using the tail vein). The mice were sacrificed 24 h after injection. Optimum cutting temperature compound (Sakura Finetek Japan, Tokyo, Japan) was used to embed liver-tissue samples prior to frozen sectioning. Cryosections of liver, which were transferred with FITC-labeled Smad decoy ODN, were examined by fluorescence microscopy.

Mice that were used in experiments were randomly divided into the three groups as follows: (1) normal control (NC) group, (2) group treated with CCl_4_ and Scr decoy ODN (CCl_4_+Scr), and (3) group treated with CCl_4_ and Smad decoy ODN (CCl_4_+Smad). Chronic liver injuries were induced by CCl_4_ intraperitoneal injection (2 mL/kg, dissolved in corn oil (at a ratio of 1:3)) three times a week.

The Scr and Smad ODN were transferred biweekly via tail vein injection, using an in vivo gene-delivery system (Mirus Bio, Madison, WI, USA) after 1 week of the first CCl_4_ injection. Eight weeks after CCl_4_ injection, the mice were sacrificed. All animals were anesthetized with Isoflurane (O_2_ 0.5 L/min, Isoflurane 2%) inhalation and Avertin (2,2,2-Tribromoethanol + 2-methyl-2-butanol + Saline Mix → After Filter 250 mg/kg intraperitoneal administration) to reduce animal pain. When the animal suffered unbearable pain, it was euthanized using CO_2_.

### 4.3. Histology Analysis

All liver tissues were fixed in 10% formalin at room temperature (RT). After fixation, the liver sections perpendicular to the anterior–posterior axis of the liver were dehydrated in graded ethanol, cleared in xylene, and embedded in paraffin. Paraffin-embedded tissues were cut into 3 μm sections and deparaffinized. Liver-tissue sections were stained with H&E and Masson’s trichrome according to standard protocol.

### 4.4. Immunohistochemical Staining

Paraffin-embedded liver sections were deparaffinized with xylene and dehydrated in gradually decreasing concentrations of ethanol. Immunohistochemical staining was performed according to the described procedure [6,38]. For immunohistochemical staining, the liver sections were incubated with a primary antibody (1:100 dilution) for 1 h at 37 °C. Primary antibodies used were as follows: antifibronection (BD Biosciences, San Jose, CA, USA) and anti-α-SMA (A2547, Sigma-Aldrich, St. Louis, MO, USA). After 3 serial washes with PBS, the sections were processed by an indirect immunoperoxidase technique using a commercial Envision System kit (DAKO, Carpinteria, CA, USA). Immunohistochemical images were viewed with an Eclipse 80i microscope (Nikon, Tokyo, Japan).

### 4.5. Immunofluorescent Staining

Paraffin-embedded liver-tissue specimens were deparaffinized with xylene and dehydrated in gradually decreasing concentrations of ethanol. The tissue samples were then placed in a blocking solution (5% bovine serum albumin in PBS) at RT for 1 h. The sections were immunostained with primary antibodies (1:500 dilution) against type I collagen (Novous Biologicals, Littleton, CO, USA), E-cadherin (Cell Signaling Technology, Danvers, MA, USA), Vimentin (BD Biosciences), and α-SMA (Abcam) at RT for 2 h. After washing, sections were incubated with the secondary antibodies (1:200 dilution) conjugated with Alexa Fluor 488 or Alexa Fluor 555 (Thermo Fisher Scientific, Waltham, MA, USA) for 30 min at 37 °C. Slides were then mounted using a Dako fluorescence mounting medium. Specimens were examined and photographed with a confocal microscope (Nikon, Tokyo, Japan).

### 4.6. Western Blot Analysis

Frozen liver tissues were lysed in cell lyticTM M lysis reagent (Sigma-Aldrich, St. Louis, MO, USA) and samples were centrifuged at 13,000 rpm for 20 min at 4 °C after incubation for 30 min on ice. The supernatant was collected, and the residual protein concentration was determined by using a Bradford assay (Bio-Rad Laboratories, Hercules, CA, USA). Total liver protein was separated on 8% to 12% Bis-Tris gels (Invitrogen), and transferred to nitrocellulose membranes (GE Healthcare, Little Chalfont, Buckinghamshire, UK). The membranes were blocked in 5% bovine-serum albumin in TBS-T (10 mM Tris, 150 mM NaCl, and 0.1% Tween-20) for 2 h at RT. Then, the membrane was probed with a primary antibody (1:1000 dilution) overnight at 4 °C. The membrane was further probed with a horseradish peroxidase-conjugated secondary antibody (1:2000 dilution) for 2 h at RT. The signals were detected using an enhanced chemiluminescence detection system (Amersham, Piscataway, NJ, USA). The primary antibodies used were antifibronectin, antivimentin (BD Biosciences), anti-p-Smad2/3, anti-Smad2/3, anti-Smad4 (Santa Cruz Biotechnology, Santa Cruz, CA, USA), anti-type I collagen (Novous Biologicals, Littleton, CO, USA), anti-α-SMA (Abcam, Cambridge, UK), anti-E-cadherin (Cell Signaling Technology, Danvers, MA, USA), and anti-glyceraldehyde-3-phosphate-dehydrogenase (GAPDH) (Cell Signaling Technology, Danvers, MA, USA).

### 4.7. qRT-PCR

Total RNA was isolated from liver tissues using TRIzol Reagent (Thermo Fisher Scientific, Waltham, MA, USA) according to the manufacturer’s methods. Reverse-transcription response was conducted by using AccuPower RT Premix and Oligo dT18 primer (Bioneer, Daejeon, Korea) according to the manufacturer’s protocols. The PCR mixtures contained 100ng of cDNA and 100pM each of forward and reverse primers. The samples were denatured at 95 °C for 10 min, followed by 45 cycles of annealing and extension at 95 °C for 20 s, 60 °C for 20 s, and 72 °C for 20 s. Real-time PCR was accomplished in a LightCycler nano System (Roche Applied Science, Mannheim, Germany) by using LightCycler DNA Master SYBR GREEN I (Roche Applied Science). Expression values were normalized to β-actin. The primer sequences used are listed below in Table 2:

### 4.8. Statistical Analysis

All data are presented as means ± SE. A Student’s *t*-test was used to assess the significance of independent experiments. Differences with *p* < 0.05 were considered significant.

## Figures and Tables

**Table 1:** Sequences of decoy used in this study.

Decoy	Sequence
Scr	5′-GAATTCAATTCAGGGTACGGCAAAAAATTGCCGTACCCTGAATT-3′
Smad	5′- GAATTCGTGTCTAGACTGAAAACAGTCTAGACAC-3′

**Table 2:** Sequences of PCR primer used in this study.

Gene	Sequence
Smad2	forward, 5′-TGCATTCTGGTGTTCAATCG -3′ reverse 5′-CGAGTTTGATGGGTCTGTGA -3′
Smad3	forward 5′-GTCAACAAGTGGTGGCGTGTG-3′ reverse 5′-GCAGCAAAGGCTTCTGGGATAA-3′
Smad4	forward 5′-TGACGCCCTAACCATTTCCAG-3′ reverse 5′-CTCCTAAGAGCAAGGCAGCAAA-3′
